# Supplementary material for: GM-CSF drives dysregulated hematopoietic stem cell activity and pathogenic extramedullary myelopoiesis in experimental spondyloarthritis
Source: Nat Commun. 2020 Jan 9;11:155. doi: 10.1038/s41467-019-13853-4 (PMC6952438; doi:10.1038/s41467-019-13853-4)
Supplement: Supplementary file 1 — Supplementary Information [file 41467_2019_13853_MOESM1_ESM.pdf]

Supplementary Information

GM-CSF drives dysregulated hematopoietic stem cell activity and pathogenic extramedullary myelopoiesis in experimental spondyloarthritis

Regan-Komito et al

This document includes 6 supplementary figures

Supplementary Figure 1

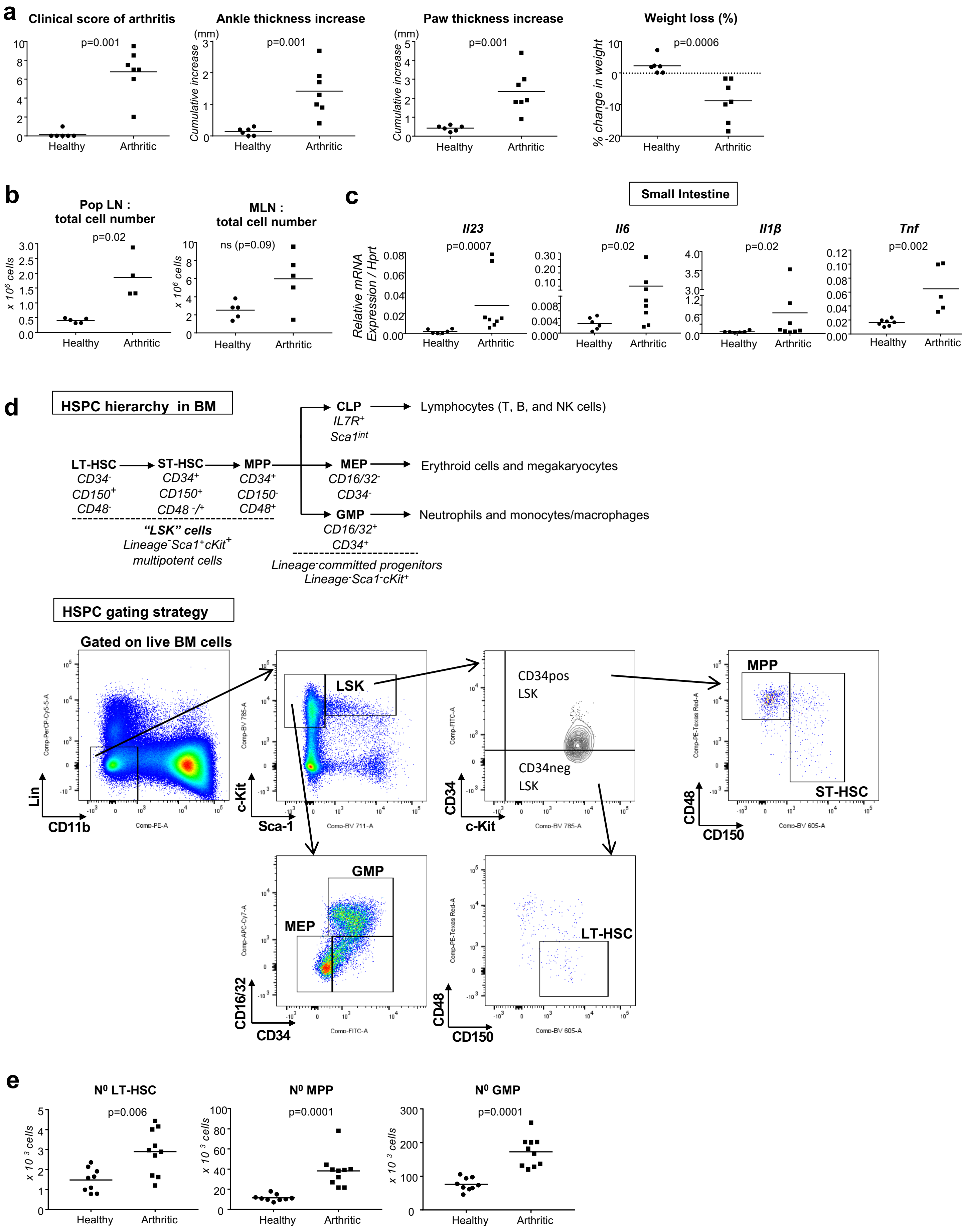

Supplementary Figure 1 continued

f

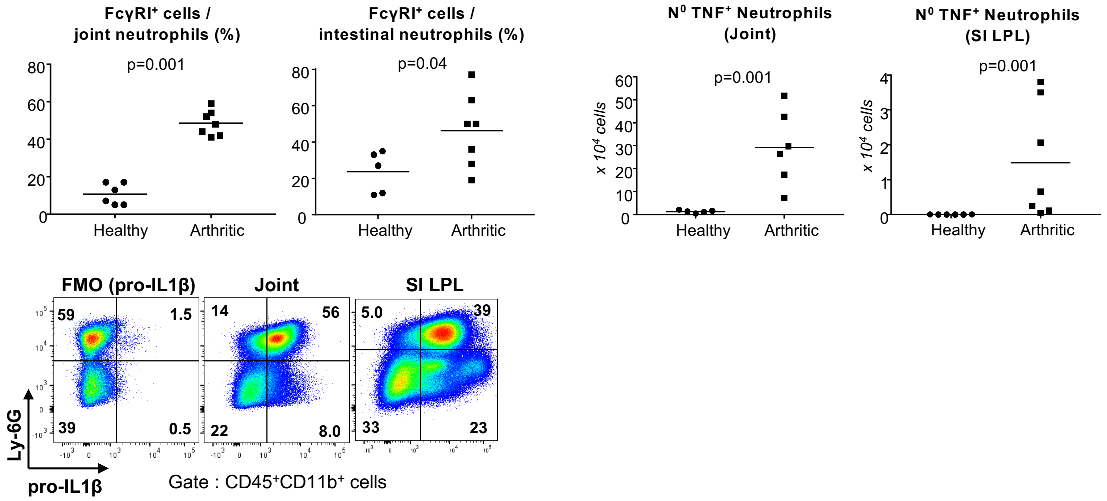

Supplementary Figure 1: Development of SpA is associated with expression of pro-inflammatory cytokines
(**a-c** and **e-f**) SKG mice were injected with curdlan IP and culled after 4-6 weeks when they developed features of SpA ("arthritic"). PBS-injected SKG mice were used as controls ("healthy").
(**a**) Measurements of disease progression in healthy SKG mice and those developing SpA. Clinical score of arthritis was derived from observation of the paws and ankles, with measurement using calipers.
(**b**) Total cell numbers obtained from popliteal or mesenteric lymph nodes.
(**c**) Expression of cytokine genes in small intestinal homogenates by RT-qPCR, standardized to expression of the reference gene *Hprt*.
(**d**) (Top) Schematic diagram of hierarchy of hematopoietic stem and progenitor cells (HSPC) in bone marrow (BM). LT-HSC: long-term hematopoietic stem cell (LSK (Lin$^-$Sca-1$^+$cKit$^+$), CD34$^-$, CD150$^+$, CD48$^-$), ST-HSC: short-term hematopoietic stem cell (LSK, CD34$^+$, CD150$^+$, CD48$^{-/+}$), MPP: multi-potent progenitor (LSK, CD34$^+$, CD150$^-$, CD48$^+$), CLP: common lymphoid progenitor (Lin$^-$, IL7Rα$^+$, Sca-1^{int,} cKit^{int}), MEP: megakaryocyte-erythroid progenitor (Lin$^-$, Sca-1$^-$, cKit$^+$, CD34$^-$, CD16/32$^-$), GMP: granulocyte-monocyte progenitor (Lin$^-$, Sca-1$^-$, cKit$^+$, CD34$^+$, CD16/32^{hi}).
(Bottom) Gating strategy for HSPCs using representative images. Of note, depending on experiments, CD11b was either included in the lineage or in a separate channel.
(**e**) Total cell numbers of LT-HSCs, MPPs, and GMPs in the BM.
(**f**) Proportion of neutrophils expressing FcγRI (CD64) and TNF by FACS among neutrophils isolated from paw joints or small intestinal lamina propria (SI LPL). Representative intracellular staining for pro-IL-1β in neutrophils from joint and SI LPL of spondyloarthritic mice, expression compared to fluorescence-minus-one (FMO) control (paw joint).
Dots represent individual mice, horizontal bars indicate mean. Data are representative of two to three independent experiments. Groups were compared using Mann-Whitney U tests.
Source data are provided as a Source Data file

Supplementary Figure 2

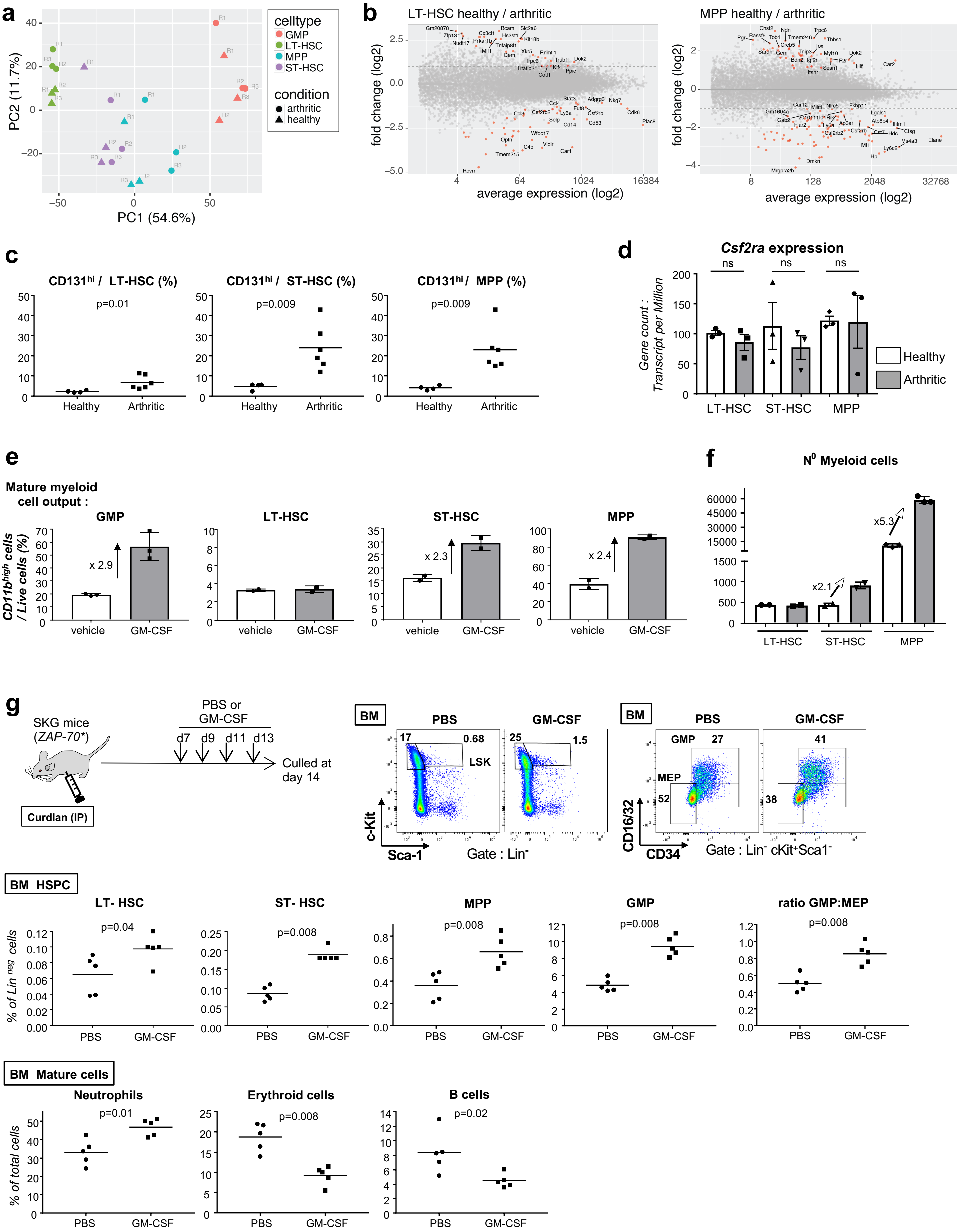

Supplementary Figure 2: HSCs and MPPs express GM-CSF-receptor and respond to it by increasing their myeloid output

(**a-b**) In 3 separate experiments, SKG mice (n=8 per group) were injected with PBS (healthy) or curdlan (arthritic) before culling after 5 weeks and sorting LT-HSCs, ST-HSCs, MPPs, and GMPs by FACS for RNA sequencing.

(**a**) Principal component analysis of samples based on genes that differed significantly between experimental groups (DESeq2 LRT test, BH adjusted $p < 0.1$).

(**b**) MA plots show genes differentially expressed in LT-HSCs (left) and MPPs (right) between healthy and arthritic mice 5 weeks after disease triggering. Red dots represent genes that are significantly differentially expressed (BH adjusted $p < 0.05$) and that have an absolute fold-change ≥ 2.

(**c**) Healthy SKG controls and arthritic mice were culled at week 5. Graphs show proportion of BM LT-HSCs, ST-HSCs and MPPs expressing the GM-CSF receptor β chain (CD131) by FACS.

(**d**) Indicated cell populations were sorted by FACS from BM of healthy and arthritic mice for RNA sequencing. Graph shows transcripts per million for the GM-CSF receptor α chain (*Csf2ra*). Bars represent mean and SEM of 3 independent biological replicates.

(**e-f**) LT-HSCs, ST-HSCs, MPPs, and GMPs were sorted by FACS for liquid culture with or without recombinant GM-CSF. After 4 days (for GMPs) or 6 days (for LT-HSCs, ST-HSCs, and MPPs), cells were analyzed. Graphs show proportion (**e**) and absolute number (**f**) of cells expressing the mature myeloid marker CD11b. Bars represent mean and SD of technical replicates.

(**g**) Experimental outline for SKG mice injected with curdlan to induce SpA then injected with PBS or GM-CSF (2 ug) IP q48h on 4 occasions, starting 7 days after injection of curdlan. Representative staining of BM cells showing frequencies of LSK ($Lin^{-}cKit^{+}Sca\text{-}1^{+}$) progenitors, GMPs, and MEPs (top). Graphs showing frequencies of HSPC populations (middle) and mature cells (bottom). Dots represent individual mice; horizontal bars represent mean.

Data are representative of two (**c**) or three (**e, f**) independent experiments. Groups were compared using Mann-Whitney U tests.

Source data are provided as a Source Data file

Supplementary Figure 3

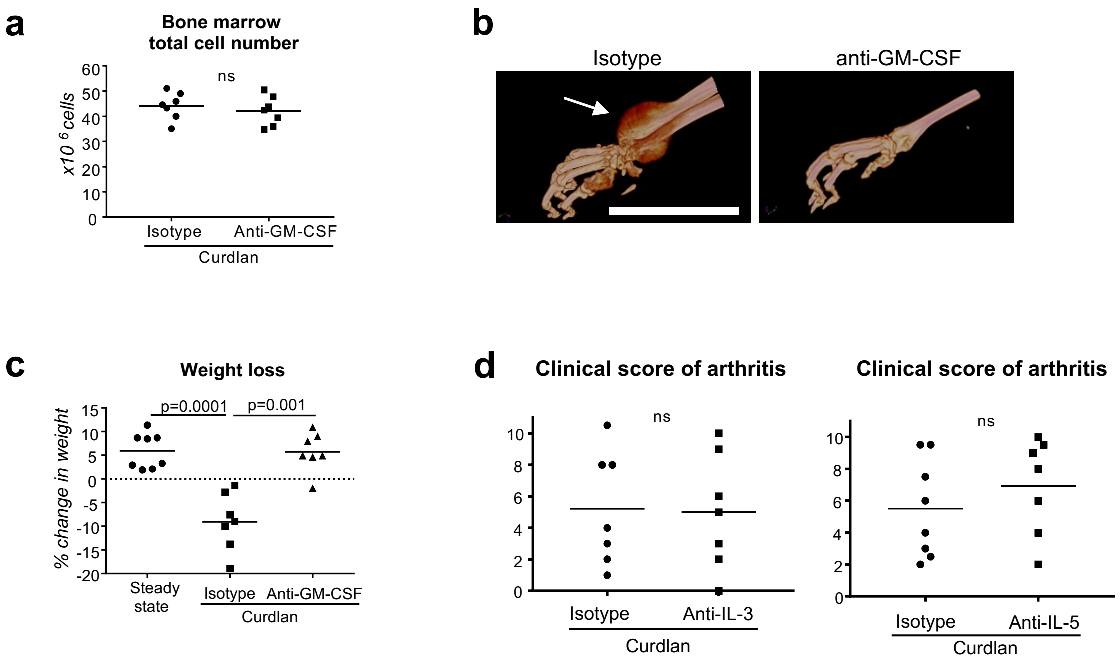

Supplementary Figure 3: **Blockade of GM-CSF prevents bone remodelling in SpA**

(**a**-**b**) SKG mice were injected with curdlan to induce SpA then injected with anti-GM-CSF antibody or its isotype IP twice weekly for 6-7 weeks.

(**a**) Total cell count of BM extracted from one tibia and one femur of each mouse. Dots represent individual mice; horizontal bars represent mean.

(**b**) Representative three-dimensional reconstructions of microcomputed tomographic (μCT) images of right front limbs. Arrow shows new bone formation characteristic of SpA. Scale bar = 1 cm.

(**c**) Change in weight. Dots represent individual mice; bars represent mean.

(d) SKG mice were injected with curdlan to induce SpA then injected with anti-IL-3 antibody, anti-IL-5 antibody, or their respective isotypes for 4 weeks. Graphs show clinical arthritis score after 4 weeks for mice injected with anti-IL-3 or isotype (left), and anti-IL-5 or isotype (right). Dots represent individual mice, horizontal bars indicate mean.

Groups were compared using Mann-Whitney U tests.

Source data are provided as a Source Data file

Supplementary Figure 4

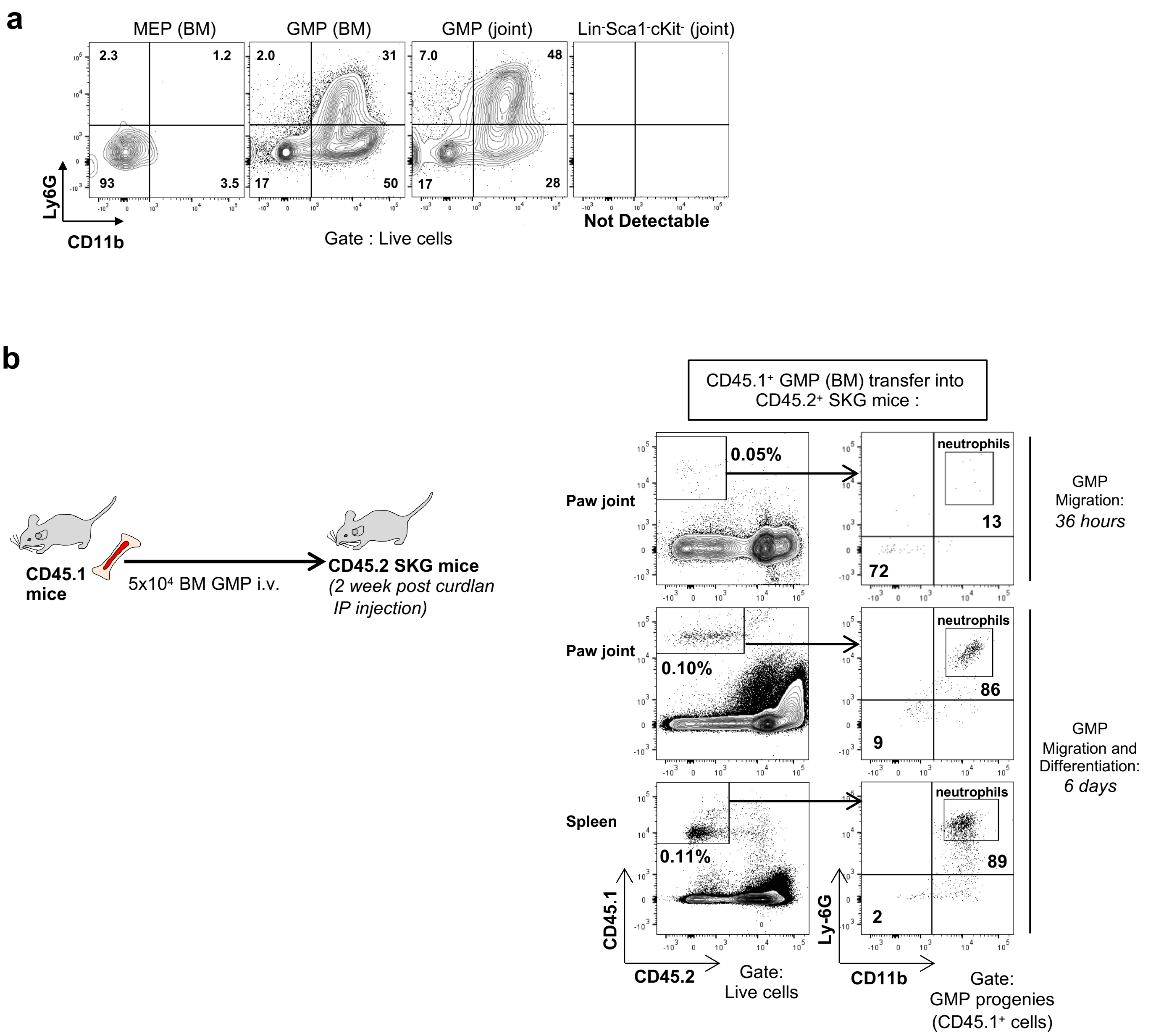

Supplementary Figure 4: GMP migrate to the joints during SpA, where they produce mature neutrophils
(**a**) MEPs, GMPs, and Lineage$^-$Sca-1$^-$cKit $^-$ cells were sorted by FACS from BM or paw joints of spondyloarthritic SKG mice. These cells were placed in liquid culture for 4 days in a medium favouring myeloid cell differentiation and then FACS stained. Images are dot plots showing frequencies of mature neutrophils (CD11b$^+$Ly6G$^+$) derived from indicated cell types. Joint Lin$^-$Sca-1$^-$cKit$^-$ progeny were not detectable. (**b**) GMPs were sorted by FACS from the BM of CD45.1$^+$ BALB/c mice and 5x10^4 cells were injected IV into CD45.2$^+$ SKG mice 2 weeks after they had been injected with curdlan to induce joint inflammation. Mice were culled 36 hours or 6 days after injection of GMPs for analysis of spleen and paws. Representative staining of cells derived from the spleen or paws are depicted, showing frequencies of mature neutrophils (CD11b$^+$Ly6G$^+$) derived from injected CD45.1$^+$ GMPs, as well as frequencies of immature cells derived from GMPs (CD11b$^-$Ly6G$^-$). Data are representative of two independent experiments.

Supplementary Figure 5

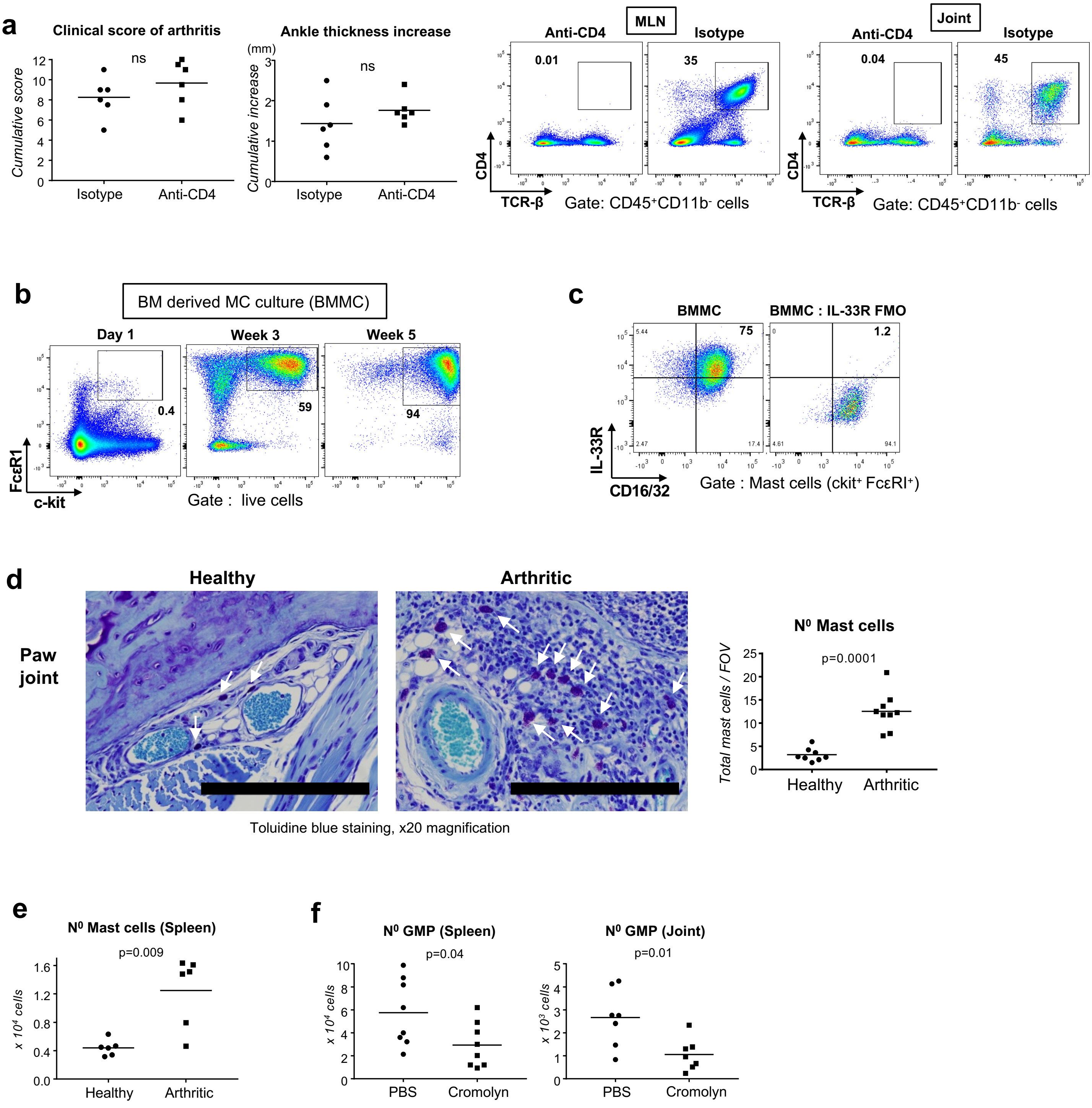

Supplementary Figure 5: Mast cell stabilization is accompanied by decreased GMP numbers in spleen and paws

(a) Mice were injected with curdlan to induce SpA and injected with anti-CD4 depleting antibodies or isotype for 4 weeks, starting 2 days prior to curdlan injection. Clinical arthritis score (far left) and change in ankle thickness (left) after 4 weeks. Points represent individual mice; bars indicate mean. Frequencies of $CD4^{+}TCR\beta^{+}$ T cells in mesenteric lymph node (MLN, right) and joints (far right) of mice treated with anti-CD4 antibodies or isotype for 4 weeks, showing the efficacy of the CD4 T cell depletion.

(**b**) Whole BM cell suspension was cultured with IL-3 for 5 weeks. Cells were stained at indicated time points to determine the frequency of mature MC ($cKit^{+}Fc\varepsilon RI^{+}$). (**c**) BM derived MC (BMMC) cells were cultured for 5 weeks with IL-3 and stained for CD16/32, and IL-33R compared to IL-33R FMO control.

Supplementary Figure 5 continued

(**d**) Representative photomicrographs of sections of paws stained with toluidine blue from healthy mice (left) and mice 4 weeks after injection of curdlan (right). White arrows indicate mast cells, x20 magnification, scale bar = 200 µm. Graph shows number of mast cells counted in field of view (FOV); 8 fields of view were counted per mouse and the mean was calculated. Graph shows number of mast cells per FOV in healthy mice and mice with SpA. Points represent individual mice; bars indicate mean. Results are pooled from 2 separate experiments.

(**e**) Mast cell numbers in the spleen of healthy and spondyloarthritic mice at week 5 quantified by FACS. Points represent individual mice, bars indicate mean.

(f) SKG mice were triggered for SpA development and then injected daily with PBS or cromolyn for 4 weeks. Graphs show absolute numbers of GMPs identified in spleen and paw joints. Dots represent individual mice; horizontal bars indicate mean.

Unless otherwise stated, groups were compared using Mann-Whitney U tests.

Data are representative of two independent experiments (**a**-**c** and **e**-**f**).

Source data are provided as a Source Data file

Supplementary Figure 6

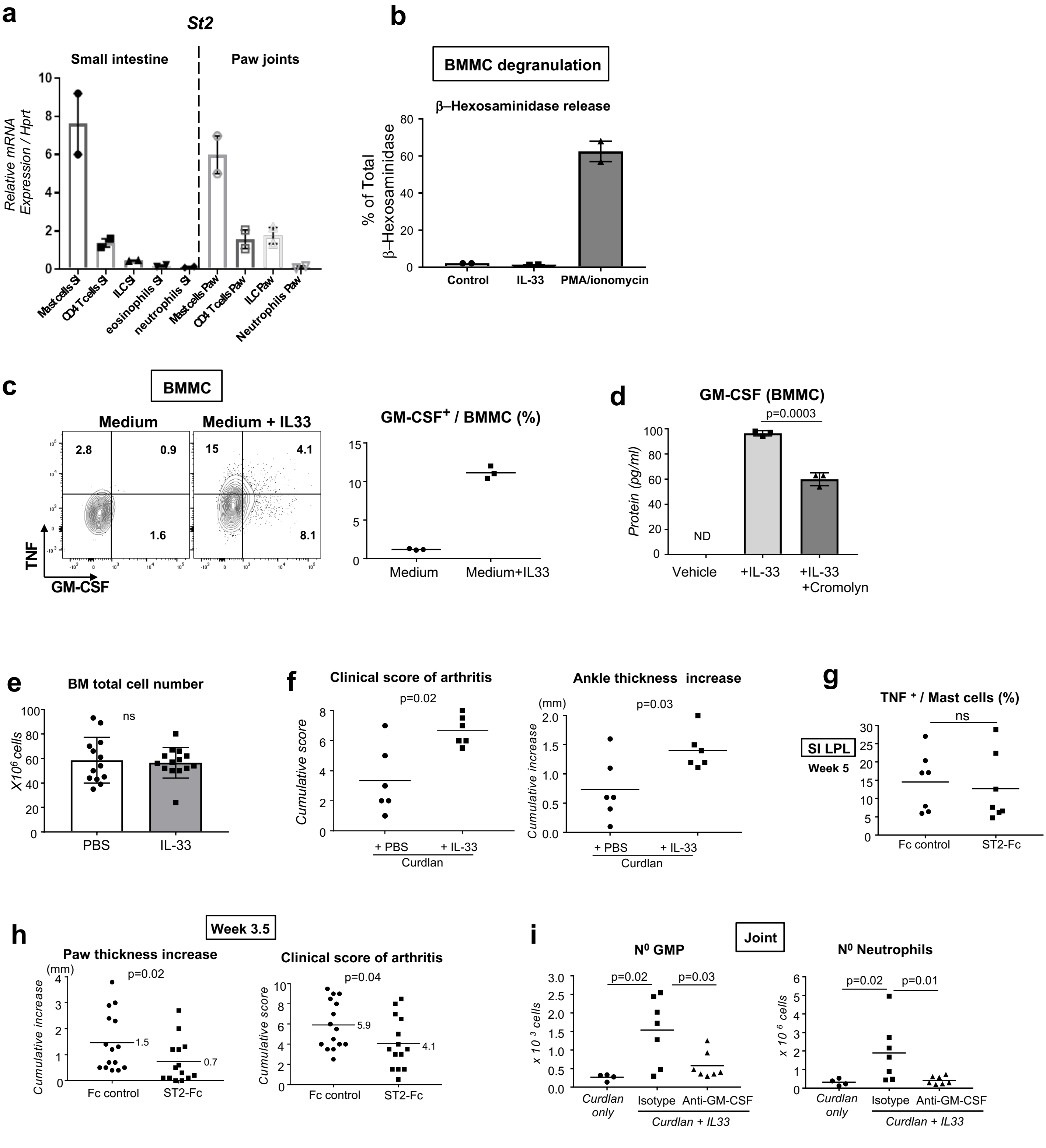

Supplementary Figure 6: Culture-derived mast cells produce GM-CSF in response to IL-33

(**a**) SKG mice were triggered for SpA development and culled after 5 weeks. MCs, innate lymphoid cells (ILC), $CD4^+$ T cells, eosinophils and neutrophils were sorted from paw and intestine by FACS for qPCR. Graphs show expression of *St2* (encoding IL-33R) relative to reference gene *Hprt*. Bars represent mean with SEM of two independent experiments.

Supplementary Figure 6 continued

(**b-d**) BM was cultured in complete medium with IL-3 for 5 weeks to derive BM mast cells (BMMCs). (**b**) BMMCs were stimulated for 30 mins with buffer, IL-33 (50 ng/ml), or PMA/ionomycin before spectrophotometric quantification of β-hexosaminidase activity in supernatant and cell lysates. Release of β-hexosaminidase from stimulated cells is expressed as a percentage of the total activity. Bars represent mean with SEM; data are pooled from two independent experiments.

(c-d) BMMCs were incubated with medium or IL-33 (50 ng/ml) for 18 hours. Where indicated, cromolyn sodium (5 mM) was added 1 hour before IL-33. (**c**) Representative images and frequencies of GM-CSF$^+$ cells among FcERI$^+$cKit^{int} activated MC. Points represent technical triplicates, bars represent mean, representative of 2 independent experiments. (**d**) Concentration of GM-CSF in supernatant of cultured cells measured by ELISA. Bars represent mean and SD of technical triplicates. ND= not detected. Groups were compared using unpaired t test.

(**e**-**f**) Male SKG mice were triggered for SpA development then injected with PBS or IL-33 twice weekly for 5 weeks, at a dose of 1 µg (**e**) or 0.5 ug (**f**) per injection. (**e**) Absolute number of BM cells derived from one femur and one tibia of each mouse (bars represent mean, n=7 mice per group). Data are pooled from two independent experiments. Points represent individual mice, bars mean with SD. (**f**) Clinical arthritis score (left) and change in ankle thickness (right) after 5 weeks. Points represent individual mice, bars indicate mean.

(**g**-**h**) Female SKG mice were injected with ST2-Fc or control Fc fragment for 5 weeks after injection of curdlan. (**g**) Frequency of TNF$^+$ cells among MC in small intestinal LPL after 5 weeks. (**h**) Change in paw thickness (left) and clinical arthritis score (right) after 3.5 weeks. Data are pooled from two independent experiments. Points represent individual mice, bars indicate mean.

(**i**) Male SKG mice were injected with curdlan, and some were injected with IL-33 twice weekly for 4 weeks, with either anti-GM-CSF antibody or isotype twice weekly. Graphs show absolute numbers of GMPs (left) and mature neutrophils (right) from paws. Points represent individual mice, bars indicate mean.

Groups compared with Mann-Whitney U test, unless stated otherwise.

Source data are provided as a Source Data file
