# Supplementary material for: GM-CSF drives dysregulated hematopoietic stem cell activity and pathogenic extramedullary myelopoiesis in experimental spondyloarthritis
Source: Nat Commun. 2020 Jan 9;11:155. doi: 10.1038/s41467-019-13853-4 (PMC6952438; doi:10.1038/s41467-019-13853-4)
Supplement: Supplementary file 3 — Reporting Summary [file 41467_2019_13853_MOESM3_ESM.pdf]

nature research

Corresponding author(s): Thibault Griseri

Last updated by author(s): Nov 25, 2019

Reporting Summary

Nature Research wishes to improve the reproducibility of the work that we publish. This form provides structure for consistency and transparency in reporting. For further information on Nature Research policies, see Authors & Referees and the Editorial Policy Checklist.

Statistics

For all statistical analyses, confirm that the following items are present in the figure legend, table legend, main text, or Methods section.

n/a	Confirmed	
☐	☒	The exact sample size (*n*) for each experimental group/condition, given as a discrete number and unit of measurement
☐	☒	A statement on whether measurements were taken from distinct samples or whether the same sample was measured repeatedly
☐	☒	The statistical test(s) used AND whether they are one- or two-sided<br>*Only common tests should be described solely by name; describe more complex techniques in the Methods section.*
☐	☒	A description of all covariates tested
☐	☒	A description of any assumptions or corrections, such as tests of normality and adjustment for multiple comparisons
☐	☒	A full description of the statistical parameters including central tendency (e.g. means) or other basic estimates (e.g. regression coefficient) AND variation (e.g. standard deviation) or associated estimates of uncertainty (e.g. confidence intervals)
☐	☒	For null hypothesis testing, the test statistic (e.g. *F*, *t*, *r*) with confidence intervals, effect sizes, degrees of freedom and *P* value noted<br>*Give P values as exact values whenever suitable.*
☒	☐	For Bayesian analysis, information on the choice of priors and Markov chain Monte Carlo settings
☒	☐	For hierarchical and complex designs, identification of the appropriate level for tests and full reporting of outcomes
☒	☐	Estimates of effect sizes (e.g. Cohen's *d*, Pearson's *r*), indicating how they were calculated

Our web collection on statistics for biologists contains articles on many of the points above.

Software and code

Policy information about availability of computer code

Data collection	See Methods section for computational analysis of RNA sequencing data. Also used were BD FACSDiva, FlowJo v10, Skyscan CT Analyzer software version 1.13.2.1, and GraphPad Prism v7.
Data analysis	See Methods section for computational analysis of RNA sequencing data. Also used were BD FACSDiva, FlowJo v10, Skyscan CT Analyzer software version 1.13.2.1, and GraphPad Prism v7.

For manuscripts utilizing custom algorithms or software that are central to the research but not yet described in published literature, software must be made available to editors/reviewers. We strongly encourage code deposition in a community repository (e.g. GitHub). See the Nature Research guidelines for submitting code & software for further information.

Data

Policy information about availability of data

All manuscripts must include a data availability statement. This statement should provide the following information, where applicable:

- Accession codes, unique identifiers, or web links for publicly available datasets
- A list of figures that have associated raw data
- A description of any restrictions on data availability

No public datasets were utilized. The figures report raw or normalised data. RNA sequencing data have been deposited in GEO (GSE126218).

Field-specific reporting

Please select the one below that is the best fit for your research. If you are not sure, read the appropriate sections before making your selection.

☒ Life sciences ☐ Behavioural & social sciences ☐ Ecological, evolutionary & environmental sciences

Life sciences study design

All studies must disclose on these points even when the disclosure is negative.

Sample size	Adequate sample sizes were used to power non-parametric statistical tests and based on pilot experiments conducted in the same laboratory.
Data exclusions	No data were excluded from analysis.
Replication	Results shown in the Figures are representative of at least 2 independent experiments or are pooled from at least 2 independent experiments, as stated in the relevant figure legends.
Randomization	Mice were randomly assigned to treatment groups.
Blinding	Experiments were not blinded but all subjective measures were complemented by objective measures of disease activity or severity.

Reporting for specific materials, systems and methods

We require information from authors about some types of materials, experimental systems and methods used in many studies. Here, indicate whether each material, system or method listed is relevant to your study. If you are not sure if a list item applies to your research, read the appropriate section before selecting a response.

Materials & experimental systems

n/a	Involved in the study	
☐	☒	Antibodies
☒	☐	Eukaryotic cell lines
☒	☐	Palaeontology
☐	☒	Animals and other organisms
☒	☐	Human research participants
☒	☐	Clinical data

Methods

n/a	Involved in the study	
☒	☐	ChIP-seq
☐	☒	Flow cytometry
☒	☐	MRI-based neuroimaging

Antibodies

Antibodies used	The following monoclonal antibodies were used for flow cytometry analysis, with the name of the clone indicated in brackets: anti-CD45 (clone 30-F11), anti-CD11b (M1/70), anti-Ter119 (Ter119), anti-Gr-1 (RB6-8C5), anti-CD3 (17A2), anti-CD4 (clone RM4-5 or clone GK1.5), anti-TCR-β (H57-597), anti-NKp46 (29A1.4), anti-CD11c (N418), anti-B220 (RA3-6B2), anti-FcER1 (MAR-1), anti-Ly-6G (1A8), anti-Ly6C (HK1.4), anti-F4/80 (BM8), anti-F4/80 (BM8), anti-CD16/32 (i.e. FcγRII/III; clone 2.4G2), anti-CD90.2 (30-H12), anti-CD117 (2B8), anti-CD34-FITC (RAM34), anti-Ly-6A/E (i.e. Sca-1; clone D7), anti-CD150 (TC15-12F12.2), anti-CD48 (HM48-1), anti-GM-CSF (MP1-22E9) and isotype control: Rat IgG2a (RTK2758), anti-TNF-α (MP6-XT22), anti-pro-IL-1β (NJTEN3), anti-IL-33Rα (i.e. ST2; clone DIH9), anti-Ki67 (16A8), anti-IL-7R (A7R34), anti-CD64 (i.e. FcγRI; clone X54-5/7.1). Antibodies were purchased from BioLegend, BD Biosciences or eBioscience. Viable cells were identified as unstained with Zombie Aqua or Zombie Green (BioLegend).
Validation	Validation was completed by the manufacturers in all cases.

Animals and other organisms

Policy information about studies involving animals; ARRIVE guidelines recommended for reporting animal research

Laboratory animals	SKG mice (BALB/c-zap70W163C) were bred and maintained under specific pathogen free (SPF) conditions. Males and females were used when aged 8-12 weeks.
Wild animals	N/A
Field-collected samples	N/A
Ethics oversight	Work involving laboratory animals was approved by the institutional Animal Welfare Ethical Review Board of the University of Oxford, under Animal (Scientific Procedures) Act 1986.

Note that full information on the approval of the study protocol must also be provided in the manuscript.

Flow Cytometry

Plots

Confirm that:

☐ The axis labels state the marker and fluorochrome used (e.g. CD4-FITC).

☒ The axis scales are clearly visible. Include numbers along axes only for bottom left plot of group (a 'group' is an analysis of identical markers).

☒ All plots are contour plots with outliers or pseudocolor plots.

☒ A numerical value for number of cells or percentage (with statistics) is provided.

Methodology

Sample preparation	See also Methods section: Single cell suspensions of desired tissues were obtained as described above. 1 to 2 x 10^6 cells were stained with zombie aqua or zombie green fixable viability dye for 15 minutes in the dark at room temperature. Cells were centrifuged and incubated with unlabelled anti-CD16/32 to block non-specific staining (unless anti-CD16/32 was in the staining panel), and were then stained with surface antibodies in PBS with 0.1% BSA for 30 minutes in the dark at 4°C. Cells were centrifuged and fixed for 15 minutes using fixation solution at 4°C. Cells were centrifuged and resuspended in PBS with 0.1% BSA and 2 mM EDTA. For intracellular staining, cells were first incubated in RPMI 1640 with 10% FCS and 5 µg/ml brefeldin A with or without PMA (5 ng/ml) and ionomycin (500 ng/ml) for 3 hours at 37 °C and 5% CO2. Cells were stained with a viability dye and for surface antigens as above for 30 minutes. After fixation, cells were then permeabilized by addition of cytofix/cytoperm solution for 15 minutes followed by addition of intracellular antibodies diluted in cytofix/cytoperm solution for 45 minutes at 4°C in the dark. Cells were centrifuged and resuspended in PBS with 0.1% BSA and 2 mM EDTA before acquisition.
Instrument	BD LSR Fortessa or BD LSRII for sample acquisition, BD FACSAria III for sorting.
Software	BD FACSDiva for acquisition and sorting, FlowJo v10 for analysis.
Cell population abundance	Absolute numbers of cells are outlined in relevant Figures.
Gating strategy	In all cases, events were initially gated by FSC and SCC, then by FSC-A and FSC-H (to exclude doublets), then by exclusion of dead events using fixable viability dyes. Subsequent gating depended on the population of interest and is outlined in the Figures.

☒ Tick this box to confirm that a figure exemplifying the gating strategy is provided in the Supplementary Information.

October 2018
